# Blood transfusions post kidney transplantation are associated with inferior allograft and patient survival—it is time for rigorous patient blood management

**DOI:** 10.3389/fneph.2023.1236520

**Published:** 2023-07-24

**Authors:** Sevda Hassan, Lisa Mumford, Susan Robinson, Dora Foukanelli, Nick Torpey, Rutger J. Ploeg, Nizam Mamode, Michael F. Murphy, Colin Brown, David J. Roberts, Fiona Regan, Michelle Willicombe

**Affiliations:** ^1^ Centre for Inflammatory Disease, Department of Immunology and Inflammation, Imperial College London, London, United Kingdom; ^2^ Imperial College Renal and Transplant Centre, Imperial College Healthcare National Health Service (NHS) Trust, Hammersmith Hospital, London, United Kingdom; ^3^ Statistics and Clinical Studies, National Health Service (NHS) Blood and Transplant, Bristol, United Kingdom; ^4^ Department of Haematology, Guys, Evelina and St Thomas National Health Service (NHS) Foundation Trust, London, United Kingdom; ^5^ Department of Haematology, Addenbrooke’s Hospital, Cambridge, United Kingdom; ^6^ Department of Clinical Nephrology and Transplantation, Cambridge University Hospitals National Health Service (NHS) Foundation Trust, Cambridge, United Kingdom; ^7^ Department of Surgery, Nuffield Department of Surgical Science, University of Oxford, Oxford, United Kingdom; ^8^ Department of Transplantation, Guys, Evelina and St Thomas National Health Service (NHS) Foundation Trust, London, United Kingdom; ^9^ National Health Service (NHS) Blood and Transplant, and Nuffield Department of Clinical and Laboratory Sciences, University of Oxford, Oxford, United Kingdom; ^10^ Haematology, National Health Service (NHS) Blood and Transplant, London, United Kingdom

**Keywords:** transfusion, kidney transplant, blood, anaemia, outcomes cRF, calculated reaction frequency DSA, donor specific antibody GFR, glomerular filtration rate

## Abstract

**Background:**

Patient Blood Management (PBM), endorsed by the World Health Organisation is an evidence-based, multi-disciplinary approach to minimise inappropriate blood product transfusions. Kidney transplantation presents a particular challenge to PBM, as comprehensive evidence of the risk of transfusion is lacking. The aim of this study is to investigate the prevalence of post-transplant blood transfusions across multiple centres, to analyse risk factors for transfusion and to compare transplant outcomes by transfusion status.

**Methods:**

This analysis was co-ordinated via the UK Transplant Registry within NHS Blood and Transplant (NHSBT), and was performed across 4 centres. Patients who had received a kidney transplant over a 1-year period, had their transfusion status identified and linked to data held within the national registry.

**Results:**

Of 720 patients, 221(30.7%) were transfused, with 214(29.7%) receiving a red blood cell (RBC) transfusion. The proportion of patients transfused at each centre ranged from 20% to 35%, with a median time to transfusion of 4 (IQR:0-12) days post-transplant. On multivariate analysis, age [OR: 1.02(1.01-1.03), p=0.001], gender [OR: 2.11(1.50-2.98), p<0.0001], ethnicity [OR: 1.28(1.28-2.60), p=0.0008], and dialysis dependence pre-transplant [OR: 1.67(1.08-2.68), p=0.02], were associated with transfusion. A risk-adjusted Cox proportional hazards model showed transfusion was associated with inferior 1-year patient survival [HR 7.94(2.08-30.27), p=0.002] and allograft survival [HR: 3.33(1.65-6.71), p=0.0008], and inferior allograft function.

**Conclusion:**

RBC transfusions are common and are independently associated with inferior transplant outcomes. We urge that further research is needed to understand the mechanisms behind the outcomes, to support the urgent development of transplant-specific anaemia guidelines.

## Introduction

Patient blood management (PBM) embodies an evidence-based, multi-disciplinary approach to minimise inappropriate blood product transfusions, in turn improving patient outcomes and reducing overall healthcare costs (1, 2). It is an international initiative endorsed by the World Health Organisation (WHO), who in 2021 announced the global implementation of PBM as an urgent healthcare intervention (3). Initially developed with surgical procedures as its focus, current PBM policies apply to all patients in whom blood product transfusions maybe required (4). Kidney transplantation presents a particular challenge to PBM, as patients with a high prevalence of anaemia are subjected to a surgical procedure which is followed by the introduction of new factors e.g. immunosuppression, which could exacerbate anaemia further (5, 6). Consequently, peri-transplant anaemia is extremely common with reported rates as high as 90% (5). Despite its common occurrence, with transplant specific contributions, guidance on post-transplant anaemia management frequently defaults to replicate protocols in the non-dialysis chronic kidney disease (CKD) population (7, 8).

A fundamental aspect to PBM is robust evidence of the risk of transfusion, which is unfortunately lacking in the setting of kidney transplantation. Such evidence cannot be extrapolated from other surgical procedures, as a unique complexity of transplantation includes the potential risk of transfusion on *de novo* allosensitisation and rejection, contributing to premature allograft loss (9–13). Although transfusion is a well-recognised cause of allosensitisation pre-transplantation, in the presence of immunosuppression in the post-transplant setting, data are less consistent (9, 10, 14, 15). However, our group has previously demonstrated that transfused transplant recipients may develop HLA antibodies against their corresponding blood donors in the post-transplant period, providing evidence that *de novo* allosensitisation may occur (9, 16).

It is unknown whether the absence of transplant specific guidelines, lack of evidence of the risk of post-transplant blood transfusions or centre specific practices contribute to the large variation in the reported transfusion rates, which range from 18.1% to 74.6% in the early post-transplant period (17, 18). However, what is clear is that to incorporate the principles of PBM in kidney transplantation, there is an urgent need for data on who is being transfused, why they are being transfused and the impact, if any, that transfusion has on outcomes.

The aim of this study is to investigate the prevalence of post-transplant blood product transfusions across multiple centres in the UK, to analyse recipient factors associated with transfusion and to compare allograft and patient survival by transfusion status.

## Materials and methods

### Setting and participants

The study was performed as part of a multidisciplinary collaboration between the National Health Service Blood and Transplant Service (NHSBT), the British Transplant Society (BTS) and the HLA matched Red Cell Working Group in the UK. Four transplant centres in the UK participated: Cambridge, London - Imperial College Renal and Transplant Centre, Oxford and London – Guy’s Hospital. The study was co-ordinated via the UK Transplant (UKT) Registry within NHSBT.

All patients who received a kidney alone transplant at one of the 4 participating centres between 1^st^ April 2016 and 31^st^ March 2017 were included. NHS Blood and Transplant employees in the blood transfusion laboratories at each centre were sent the relevant list of local transplant recipients via the UKT Registry, who then confirmed transfusion status. In the UK, the Medicines and Healthcare products Regulatory Agency (MHRA) expects blood product traceability, that is the ability to trace each individual product from the donor to its destination in 100% of cases. Each laboratory collected the same baseline information, which included: the transfusion status (including one month before, to 12 months post-transplant), the date of the first transfusion, the type of transfusion and the total amount of units received within the given timeframe. Of note, no patient was transfused in the month prior to transplant. The responses from each haematology laboratory were then returned to NHSBT for amalgamation, allowing the transfusion cohort to be assessed in the context of nationally collected data, which includes recipient demographics (gender, age, ethnicity, cause of end-stage renal disease (ESRD), pre-emptive status, level of sensitisation and mismatch level), donor information (deceased or living, standard or extended criteria, donor age, cold ischaemic time) and outcomes (delayed graft function, allograft function reported as estimated glomerular filtration rate (GFR), graft survival and patient survival).

The analysis was performed within NHSBT as part of a service evaluation, and as used routinely collected data, institutional approval was obtained. The study has been reported in line with the STROBE (Strengthening the Reporting of Observational studies in Epidemiology) guidelines for observational studies.

### Statistical analysis

Differences in donor and recipient characteristics between transfused and non-transfused groups were examined using the Kruskal-Wallis test or the Chi-square test. Number and percentage of missing variable data were detailed in the appropriate tables and complete case analysis was employed. Kaplan-Meier survival estimates were used to demonstrate death censored graft survival and patient survival; univariate differences between groups were examined using the log-rank test. A binary logistic regression model was used to report independent donor and recipient characteristics associated with odds of transfusion. A Cox proportional hazards model was used to assess the risk-adjusted association between transfusion status and death censored allograft and patient survival; risk adjusted for dialysis at transplant, waiting time, recipient age, cold ischaemic time, donor age. Two-sided tests were conducted and *p* < 0·05 was considered statistically significant. Data were analysed using IBM SPSS Statistics for Macintosh version 25 (IBM).

## Results

Seven hundred and twenty patients received a kidney transplant across the 4 centres between 2016 and 2017. Of these, 221/720 (30.7%) were transfused; 189 (26.3%) received a red blood cell (RBC) transfusion alone, 7 (1.0%) platelets alone and 25 (3.5%) received both blood and platelet transfusions. There was inter-centre variation in the proportion of patients transfused, ranging from 20% to 35%, p=0.005. The median time to transfusion was 4 (IQR:0-12) days post-transplant, and the median number of units transfused was 2 (IQR:2-5) units of blood and 1 (IQR:1-3) pool of platelets.

### Kidney donor characteristics associated with transfusion

Kidney donor characteristics associated with transfusion included deceased donor status and increasing donor age, **Table 1**. One hundred and seventy-nine (36%) of patients who received a transplant from a deceased donor were transfused compared with 42 (19%) of patients receiving a transplant from a living donor, p<0.0001. The average donor age in the transfused group was 56 (IQR:46-68) years compared with 52 (IQR:40-63) years in the non-transfused group, p=0.0003. Analysing the characteristics of the deceased donors separately, neither cause of death or extended donor criteria status impacted on risk of transfusion, whilst recipients who were transfused were more likely to receive a transplant from a donor with a higher UK kidney donor risk index (UKKDRI) than patients who were not transfused, with a score of 1.46 (IQR:1.04 – 1.86) and 1.28 (IQR:1.00 – 1.53) respectively, p=0.001 (19).

**Table 1:** Demographics of donors and respective recipients receiving a transfusion.

Variable		Transfusion N=221 (%)	No Transfusion N=499 (%)	p value
Donor Characteristics				
**Donor Type**	Deceased Donor DBD DCD Living	179 (81) 81 (37) 98 (44) 42 (19)	319 (64) 175 (35) 144 (29) 180 (36)	<0.0001
**Donor Gender**	Male Female	114 (52) 107 (48)	257 (51) 242 (49)	0.9
**Donor age**	Years (Median IQR)	56 (46 – 68)	52 (40 – 63)	0.0003
**Donor Ethnicity**	White Non-white Unknown	194 (88) 23 (10) 4 (2)	440 (88) 54 (11) 5 (1)	0.7
Donor Hypertension*	No Yes Unknown	72 (40) 104 (58) 2 (2)	92 (29) 223 (70) 3 (1)	0.03
**Donor cause of** Death*	CVA RTA Miscellaneous	86 (39) 1 (<1) 92 (42)	166 (33) 2 (<1) 151 (30)	0.7
ECD status*	No Yes	86 (48) 93 (52)	180 (56) 139 (44)	0.07
UKKDRI*	Median IQR	1.46 (1.04 – 1.86)	1.28 (1.00 – 1.53)	0.001
Recipient Characteristics				
**Gender**	Male Female	121 (55) 100 (45)	348 (70) 151 (30)	<0.0001
**Ethnicity**	White Non-white Unknown	120 (54) 96 (43) 5 (2)	340 (68) 139 (28) 20 (4)	0.0002
**Age**	Years (Median IQR)	55 (44 – 64)	50 (37 – 60)	<0.0001
**Primary renal disease**	Glomerulonephritis Pyelonephritis/TIN Miscellaneous Polycystic kidneys Hypertension/RVD Diabetes Unknown	50 (23) 17 (8) 37 (17) 26 (12) 18 (8) 23 (10) 50 (23)	102 (20) 34 (7) 91 (18) 71 (14) 25 (5) 55 (11) 121 (24)	0.7
**Transplant waiting time**	Days (Median IQR)	563 (289 – 1102)	397 (168 – 861)	0.001
**Pre-emptive transplant**	No Yes	190 (86) 31 (14)	366 (73) 133 (27)	0.0002
**Graft number**	First graft Re-graft	181 (82) 40 (18)	427 (86) 72 (14)	0.2
**cRF at time of transplantation**	0 – 15 16 – 84 85 - 100	147 (67) 47 (21) 27 (12)	354 (71) 115 (23) 30 (6)	0.002
**HLA mismatch**	Level 1 Level 2 Level 3 Level 4	14 (6) 58 (26) 111 (50) 38 (17)	47 (9) 141 (28) 248 (50) 63 (13)	0.2
**Cold Ischaemic Time**	Hours (Median IQR)	11.5 (8.8 – 16.4)	9.5 (4.8 – 14.5)	<0.0001
**Graft Function**	Immediate Delayed Primary non-function Unknown	132 (60) 69 (31) 6 (3) 14 (6)	424 (85) 53 (11) 8 (2) 14 (3)	<0.0001

*Deceased donors only; DBD, donation after brain death; DCD, donation after cardiac death; CVA, cerebrovascular accident; RTA, Road traffic accident; ECD, extended criteria donor; UKKDRI, UK kidney donor risk index; IQR, interquartile range; cRF, calculated reaction frequency; TIN, interstitial nephritis; RVD, renovascular disease; IQR, interquartile range; Level 1 000 mismatch, Level 2 mismatch 0DR and 0/1B, Level 3 mismatch (0DR +2B) or (1DR +0/1B), Level 4 (1DR + 2B) or 2DR.

### Recipient characteristics associated with transfusion

Recipient variables which were found to be associated with transfusion on univariate analysis included gender, ethnicity, age at transplant, pre-emptive status, time on the wait list and degree of HLA sensitisation, **Table 1**. Females were more likely to be transfused than males, with 39.8% of females and 25.8% of males transfused respectively, p<0.0001. The transfusion group were more likely to be older, with a median age of 55 (IQR:44–64) years compared with 50 (IQR:37–60) years in the non-transfusion group, p<0.0001. Patients from white ethnic backgrounds were less likely to be transfused than those from non-white backgrounds, with a transfusion prevalence of 26.9% and 40.9% respectively, p=0.0001. Patients who had a pre-emptive transplant were also less likely to be transfused than those who did not receive a pre-emptive transplant, with a prevalence of 19.1% compared with 33.6% respectively p=0.0002. Highly sensitised patients, defined as having a calculated reaction frequency (cRF) of >85% also had a high risk of transfusion, with 47.4% of highly sensitised patients compared with 29.3% non-highly sensitised patients, p=0.005, being transfused.

The median cold ischaemic time was longer in the transfused group compared with the non-transfused group at 11.5 (IQR:8.8–16.4) versus 9.5 (IQR:4.8–14.5) hours respectively, p<0.0001. The proportion of patients with immediate graft function was also less in the transfused group at 132 (60%) compared with the non-transfused group in whom 424 (85%) had immediate graft function, p<0.0001.

### Multivariable analysis of donor and recipient factors associated with transfusion

On multivariate analysis, donor and recipient factors which were associated with transfusion included recipient age, gender, ethnicity, and dialysis dependence at the time of transplant, whilst the only independent kidney donor characteristics associated with the need for a post-transplant transfusion was a history of hypertension, **Table 2**. Older recipients were more likely to be transfused, OR: 1.02 (95% CI 1.01-1.03), p=0.001; whilst female gender and non-white ethnicity were also associated with transfusion, OR: 2.11 (95% CI 1.50-2.98), p<0.0001 and OR: 1.28 (95% CI 1.28-2.60), p=0.0008 respectively. Patients who received a pre-emptive transplant were also less likely to be transfused compared with those patients who were established on dialysis at the time of transplantation, OR: 1.67 (95% CI 1.08-2.68), p=0.02.

**Table 2:** Binary logistic regression model for transfused patients after adult kidney alone transplants in the UK.

Factor	Level	Number included	Odds Ratio	95% CI	p-value
**Recipient Gender**	Male Female	469 251	1.00 2.11	1.50-2.98	<0.0001
**Recipient Ethnicity**	White Non-white Unknown	459 237 27	1.00 1.83 0.87	1.28-2.60 0.1-2.49	0.0008 0.8
**Recipient Age**	Every year	720	1.02	1.01-1.03	0.001
**Dialysis at transplantation**	No Yes	164 556	1.00 1.67	1.08-2.68	0.02
**Donor Hypertension**	No Yes Unknown	327 164 229	1.00 1.55 0.68	1.03-2.34 0.45-1.04	0.03 0.08

Further details on indications for transfusion was available from one centre, and maybe found in the **Supplemental Information**. From these data, of 45 blood transfusion episodes, only 8 (18%) occurred intra-operatively.

### Allograft outcomes

**Figure 1:**
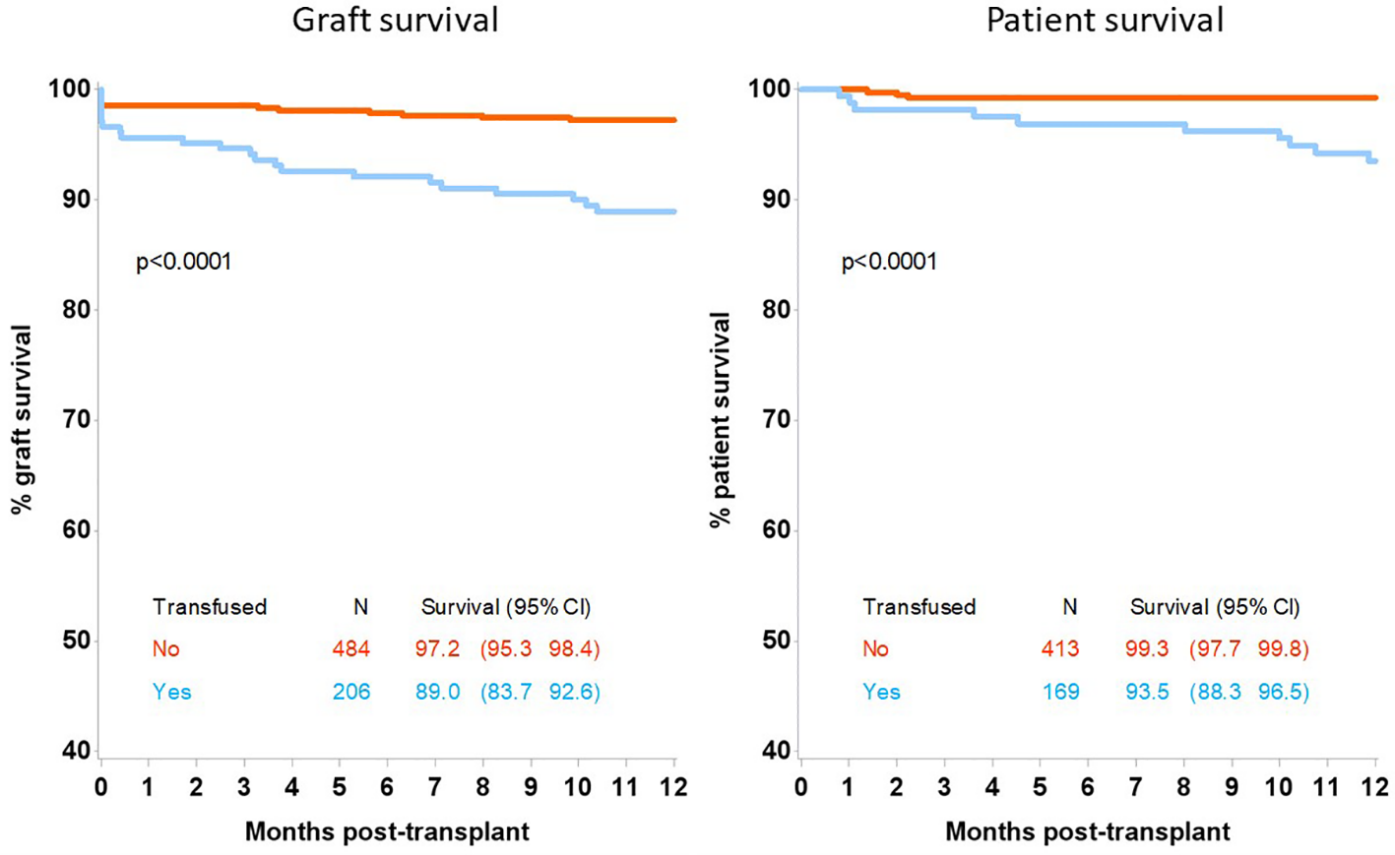
12-month graft and patient survival following kidney only transplant by transfusion status.

Patient survival at 12 months post-transplant was inferior in the transfused group at 93.5% (95%CI 88.3-96.5) compared with 99.3% (95%CI 97.7-99.8) in the non-transfused group, p<0.0001, **Figure 1**. Allograft survival was also inferior in patients who were transfused compared with non-transfused patients with a survival of 89.0% (95% CI 83.7-92.6) and 97.2% (95%CI 95.3-98.4) respectively, p<0.0001. Renal transplant function, determined by estimated GFR was inferior at 3 months and 12 months in the transfused group at 39 (IQR:22-57) and 38 (IQR:24-57) mls/min compared with the non-transfused patients, who had a GFR of 52 (IQR:40-67), p<0.0001 and 53 (IQR:42-65) mls/min, p<0.0001 respectively, **Table 3**.

**Table 3:** Comparison of transplant function at 3 and 12 months post-transplant.

Time post-transplant		Transfusion Type				p value
		No Transfusion	Blood Alone	Platelets Alone	Blood and Platelets	
**3 months**	Number of patients	474	152	7	19	<0.0001
	eGFR* (Median IQR)	52 (40 – 67)	41 (31-57)	37 (29-51)	29 (22-43)	
**12 months**	Number of patients	432	149	5	15	<0.0001
	eGFR* (Median IQR)	53 (42 – 65)	42 (32-57)	27 (24-36)	31 (24-38)	

*estimated glomerular filtration rate in mls/min.

**Figure 2:**
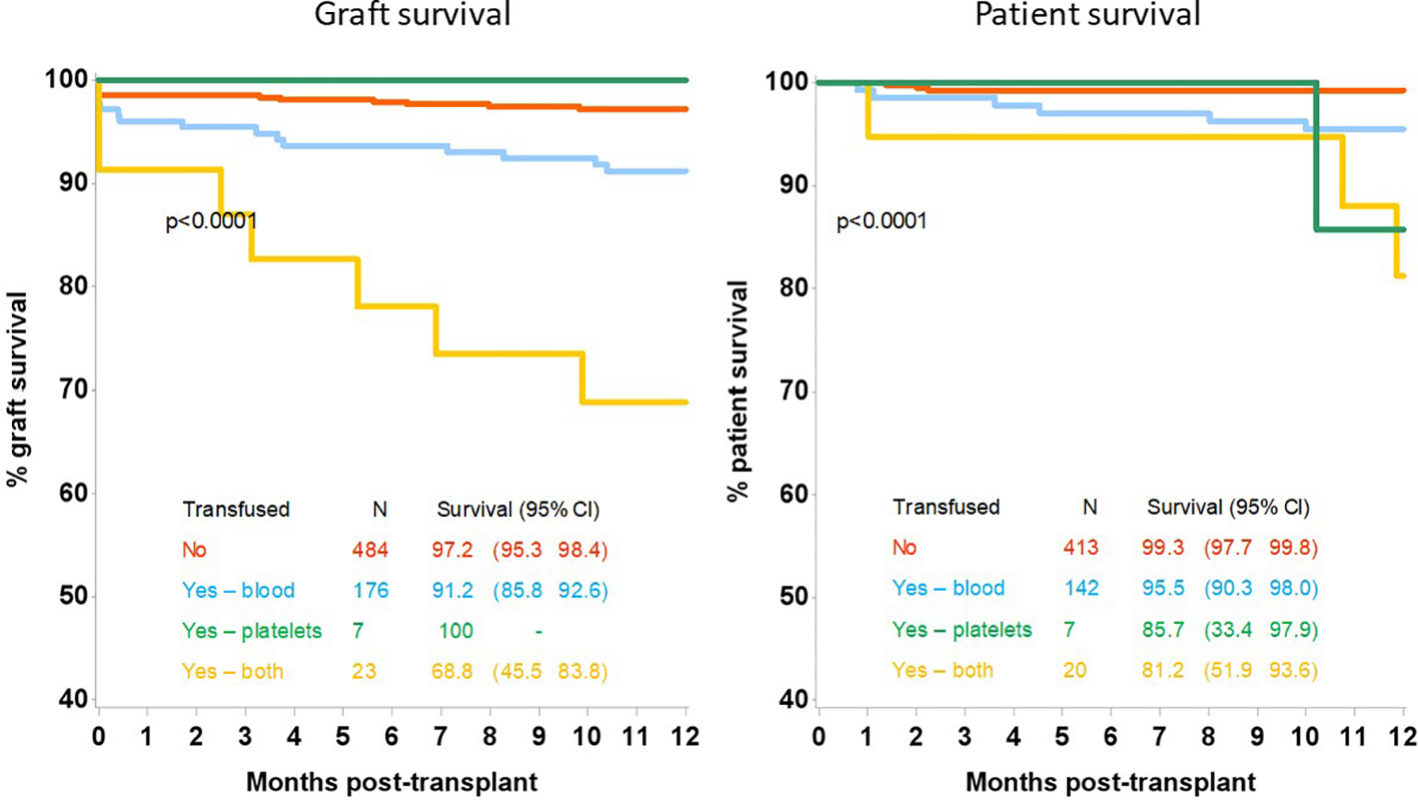
12-month allograft and patient survival following kidney only transplant by transfusion type.

Analysing the survival outcomes by type of transfusion received, patients who had both blood and platelets transfused had the worst 12 month patient and allograft survival at 81.2% (95%CI 51.9-93.6) and 68.8% (95%CI 45.5-83.8) respectively; which was significantly worse than patients receiving blood transfusions alone, who had a 12 month patient and allograft survival of 95.5% (95%CI 90.3-98.0), p=0.03 and 91.2% (95%CI 85.8-92.6) respectively, p=0.0015, **Figure 2**. Recipient GFR at 3 months and 12 months was also significantly different depending on the type of transfusion received, **Table 3**. At 3 months post-transplantation, GFR in recipients not transfused, transfused with blood alone, platelets alone or both blood and platelets was 52 (IQR:40–67), 41 (IQR:31-57), 37 (IQR:29-51) and 29 (IQR:22-43) mls/min respectively, p<0.0001; whilst at 12 months the corresponding GFR was 53 (IQR:42–65), 42 (IQR:32-57), 27 (IQR:24-36) and 31 (IQR:24-38) respectively, p<0.0001.

The final analysis involved assessing the independent impact of transfusion on the 12-month patient and allograft survival by performing a risk-adjusted Cox proportional hazards model. Variables adjusted in the model included the need for dialysis at the time of transplant, the transplant wait time, recipient age, kidney donor age and cold ischaemic time. Patient survival at 12 months was inferior in recipients who had received a post-transplant transfusion compared with non-transfused patients, HR 7.94 (95%CI 2.08-30.27), p=0.002, **Table 4**. Considering patients who received a blood transfusion alone, inferior survival was maintained compared with those patients who were not transfused, HR 5.98 (95%CI 1.42-25.10), p=0.01. Repeating the analysis for death censored allograft survival at 12 months, transfusion was associated with inferior allograft graft, HR: 3.33 (95%CI 1.65-6.71), p=0.0008, **Table 4**. Risk of allograft loss was still significantly increased when considering patients who received blood only, HR: 2.69 (9% CI 1.26-5.72), p=0.01; but was highest in patients who received both blood and platelets, HR: 11.13 (95%CI 4.26-29.08), p<0.001.

**Table 4:** Risk-adjusted Cox Proportional hazards one-year patient and allograft survival model after adult kidney only transplant.

Factor		Number included	HR	95% CI	p-value
Patient survival					
**Transfused**	No Yes	413 169	1.00 7.94	2.08-30.27	0.002
**Transfusion Type**	None Blood Blood and Platelets Platelets	413 142 7 20	1.00 5.98 9.15 22.68	1.42-25.10 0.93-90.03 3.91-131.60	0.01 - -
Allograft survival					
**Transfused**	No Yes	484 206	1 3.33	1.65 – 6.71	0.0008
**Transfusion Type**	None Blood Blood and Platelets Platelets	484 176 23 7	1 2.69 11.13 -	1.26 – 5.72 4.26 – 29.08 -	0.01 <0.001 -

Risk adjusted for dialysis at transplant, waiting time, recipient age, cold ischaemic time, donor age.

## Discussion

In this multicentre study we have demonstrated that post-transplant transfusions are common, with significant variation in rates across the different centres. Multivariable analysis of clinical characteristics associated with transfusions identified that recipient characteristics had a greater impact on transfusion risk than kidney donor characteristics. Furthermore, we have shown that transfusions, which mainly occur in the early post-transplant period are associated with inferior allograft function, allograft survival and patient survival at 12 months post-transplant, which is maintained even after adjusting for clinical characteristics recognised to be associated with poor allograft outcomes. Therefore, independent of the potential impact on allosensitisation, these data provide evidence that the transplant community needs to address these inequalities and explore the mechanisms behind the adverse outcomes to inform interventions which may improve patient health and transplant longevity.

The underlying principles or 3 pillars of PBM relate to the detection and management of anaemia, minimisation of blood loss including optimisation of coagulation, and optimising patient specific tolerance of anaemia (3). For patients undergoing kidney transplantation, each pillar requires the application of renal and transplant specific considerations. From optimisation of pre-transplant anaemia, to circumventing the dichotomy of increased bleeding and thrombotic risks, to balancing adverse effects of immunosuppression, prophylactic anti-microbials and infection (6, 20, 21). Despite this, collective evidence and therefore clinical guidelines are lacking, which is likely to be contributing to the variation of management of anaemia and transfusion rates (22, 23).

We found that the requirement for blood transfusions predominantly occurs in the early post-transplant period, which is supported by other reports (5, 9, 24). Although blood loss during surgery will contribute to the need for early transfusion, our data suggests that intraoperative transfusions occur less frequently than in the early post-transplant period. By measuring erythropoietin (EPO) levels post-transplant, it has been shown that EPO follows a bimodal distribution post-transplantation, with sustained levels from 28 days in the setting of primary graft function (25). Therefore, it is the first couple of weeks where efforts to optimise anaemia management needs to be prioritised in the first instance.

Recombinant erythropoietin (rEPO) and iron therapy have been and remain the cornerstones of the treatment of renal anaemia. However, rEPO use is not without risk, with data from clinical trials demonstrating an increased risk of cardiovascular events when normal haemoglobin or haematocrit thresholds are passed (26–29). Moreover, in one retrospective observational study, rEPO use to increase haemoglobin levels in renal transplant recipients was associated with increased mortality risk, suggesting careful dosing is required (30). In the past few years, iron therapy has had a renaissance, with clinical studies showing the benefit of more aggressive iron therapy in renal patients (CKD and dialysis), improving clinical outcomes whilst reducing rEPO use and blood transfusions, in the absence of increased infection rates (31, 32). In the setting of transplantation consideration must also be given to a recent meta-analysis of the administration of intravenous iron, which suggested its use to be associated with enhanced infection risk (33). Although this study was limited by standardised definitions of infection, the role of iron in the post-transplant period, when infection risk is high is not clear. Irrespective of its role in the post-transplant period, adequate iron therapy in the pre-transplant setting is of upmost importance, and proactive iron for those patients on the wait-list now seems a sensible approach to optimise patients prior to transplantation (32). In addition to administering adequate iron supplementation, it is also imperative to investigate, where indicated, occult causes of blood loss and iron deficiency; together with other vitamin deficiencies and causes of anaemia prior to transplantation.

Even in patients fully optimised pre-transplant, the need for blood transfusions will never be eradicated, and although not addressed in this study, the medium- and long-term impact of transfusion induced allosensitisation in kidney transplant risk is still an area of concern that warrants further investigation independently. The transplant community recognises that the prevention of allosensitisation is vital, given the lack of effectiveness of therapeutic interventions, both for desensitisation and treatment of antibody mediated rejection. We acknowledge that an issue remains of the conflicting evidence of the relevance of post-transplant transfusions on *de novo* HLA and donor specific antibody (DSA) development in the literature (10, 11, 13–15, 17, 34, 35). However, the major limitation of reported series include small sample size, short duration and the clinical characteristics of the patient cohorts studied. We have previously shown that transplant recipients can develop HLA antibodies against blood donors, and if the blood and transplant donor shared HLA mismatches with the transplant recipient, then clinical outcomes were worse (9). We hypothesise, that in the UK, where the majority of the blood and transplant donors are of white ethnicity, but there is a disproportionately high number of non-white kidney transplant recipients, this may contribute to the greater impact of allosensitisation (36, 37). Given we have reported that the risk factors for transfusion includes non-white ethnicity, and that it is recognised that non-white transplant recipients have inferior allograft survival compared with white transplant recipients in multi-ethnic communities, this again supports the call for research in this area.

This study would have been strengthened with additional data related to immunosuppression type, rejection and *de novo* DSA. Given the median time to transfusion was 4 days post-transplant, we did not employ a time-varying coefficient model, which is recognised as a further potential limitation. Key data on haemoglobin and iron levels are unavailable, and further information on causes of graft loss and death, may have helped to delineate cause from association. However, the multicentre nature of this study has highlighted the high frequency of transfusions is a common complication, and the impact of graft and patient survival at 12 months more consequential than previously considered. It needs to be acknowledged that this is not the first study to show an association of transfusion with graft and patient outcomes, however, it’s strength is the multicentre approach, incorporating analyses on both donor and recipient characteristics using granular data to report on multiple clinical outcomes including allograft function (9–13, 18, 38).

To summarise, PBM applied to general surgical procedures has been shown to reduce the need for transfusions and improve patient outcomes by lowering morbidity and mortality (1, 2). In the field of kidney transplantation, where patients at high risk of anaemia in whom the potential consequences of transfusion could be life long, evidence to aid PBM is relatively scarce. We have shown, in this multi-centre study, that blood transfusions are common and are independently associated with inferior allograft and patient outcomes at one-year post-transplant. We urge that further research is urgently needed to understand the mechanisms behind the outcomes, to help shape and develop transplant specific anaemia guidelines, responding to a call to action for PBM implementation by the WHO.

## Data availability statement

The data analysed in this study is subject to the following licenses/restrictions: The datasets generated and analysed during the current study are not publicly available due to analysis being performed as part of a service evaluation within NHSBT of routinely collected clinical data. Requests to access these datasets should be directed to m.willicombe08@imperial.ac.uk.

## Ethics statement

The data in this manuscript was acquired as part of routine clinic care, and analysed as part of a service evaluation, and obtained institutional approval from NHSBT under clinical governance arrangements, which does not need research ethics committee review under Health Research Authority guidance (39). As the data were obtained from routinely collected clinical information by the care teams, individual consent was not required.

## Author contributions

SH, MW, FR, CB and DR conceptualised the study. All authors contributed to the research methodology. SR, DF, MM, DR, FR and LM participated in data acquisition. LM performed the data analysis. SH, MW, FR, DR wrote the initial draft. All authors reviewed and edited the manuscript.
