# Supplementary material for: Blood transfusions post kidney transplantation are associated with inferior allograft and patient survival—it is time for rigorous patient blood management
Source: Front Nephrol. 2023 Jul 24;3:1236520. doi: 10.3389/fneph.2023.1236520 (PMC10479650; doi:10.3389/fneph.2023.1236520)
Supplement: Supplementary file 1 [file Table_1.docx]

**Supplemental Information**

**Sub-study analysis of indications for transfusions**

Data on indications for transfusion was available for one of the centres. During the study period, 60/172 (35%) transplant recipients from this centre had a transfusion. The breakdown of the components given in this period can be seen in **Table 1**. Transfusions occurred early post-transplant, at a median time point of 2.5 days (IQR: 0-14.25). The median number of blood and platelet transfusions received was two (IQR: 2-5) and zero (IQR: 0-1) per patient respectively. There was no difference in haemoglobin at transplantation between the transfused and non-transfused cohorts, 117g/l (IQR: 107-127) and 116g/l (IQR: 108-125) respectively. The mean haemoglobin at blood transfusion was 82 ± 16g/l.

To understand the indications behind the transfusion, they were regrouped according to transfusion episodes. Thereby, if blood and platelets were given within the same week – then that was counted as one episode. This occurred in nine cases and apart from one case, where the components were transfused seven days apart, the others both received the components at the same time. When the blood and platelets were given at different time points (beyond a week from each other), then they were counted as individual episodes. This therefore resulted in 45/65 (69%) blood only transfusion episodes, 11/65 (17%) platelet only transfusion episodes and 9/65 (14%) blood and platelet combined transfusion episodes. The indications for these transfusions are listed in **Table S1**.

**Table S1 Indications for transfusion episodes**

| **Blood only transfusion episode** |
| --- |
| 1. Delayed Graft Function (DGF): 10/45 (22%) 2. Post-transplant anaemia (PTA); previously on EPO: 8/45 (18%) 3. Intra-operative transfusion for bleeding: 8/45 (18%) 4. Biopsy ± DGF: 7/45 (16%) 5. (PTA) Bleeding; transfused post-op: 5/45 (11%) 6. (PTA) Rejection: 2/45 (4%) 7. Other surgical complications: 2/45 (4%) 8. Other medical complications: 2/45 (4%) 9. Symptom control: 1/45 (2%) |
| **Combined Blood and Platelet transfusion episode** |
| 1. Post-operative bleed*: 6/9 (67%) 2. Intra-operative bleed: 1/9 (11%) 3. Procedure (Percutaneous Nephrostomy): 1/9 (11%) 4. Unknown: 1/9 (11%) |
| **Platelet only transfusion episode** |
| 1. Intra-operative bleed with thrombocytopenia: 5/11 (45%) 2. Post-operative thrombocytopenia ± DGF: 4/11 (36%) 3. Biopsy: 2/11 (18%) |

*Two patients had an additional comorbidity, which complicated the bleed – underlying chronic liver disease; Bone Marrow Failure
